# Monthly Summary of Medical Journalism

**Published:** 1860-05

**Authors:** O. C. Gibbs

**Affiliations:** Frewsburg, N. Y.


### ECLECTIC DEPARTMENT,

### AND SPIRIT OF THE MEDICAL PERIODICAL PRESS.

*Monthly Summary of Cotemporary Medical Journalism.* By O. C. Gibbs, M.D., Frewsburg, N. Y.

*Dysentery.*—In the *Lancet and Observer* for March, Prof. E. S. Cooper, of San Francisco, has an article upon the treatment of dysentery. He thinks very highly of ipecacuanha as a remedy in such cases. He gives emetic doses in the early stages, and small doses, with opium and acetate of lead, frequently repeated, in the more advanced stages: "say one-sixteenth of a grain of each, repeated every ten, fifteen, or twenty minutes." We have previously referred to the treatment of dysentery with large doses of ipecacuanha, which, so far as we know, was first proposed by E. S. Docker, Esq. Since we have learned the powers of Epsom salts, elixir vitriol, and morphine, in appropriate combination, over this disease, we have had no disposition to try anything new. Prof. Cooper makes one remark that is perhaps worthy of further consideration. He says, "I have found many cases in which ipecacuanha combined with extract of gentian, in the proportion of one-half grain of the former to one of the latter, every hour, acted almost like a charm in the advanced stages of protracted cases, where other remedies had only produced a palliation of the symptoms."

We must protest against the consideration of *dysentery and diarrhœa* together, as does Prof. Cooper, as though they were allied diseases, requiring identical, or, at least, similar treatment. Cathartics, of a certain character, are always appropriate in a pure case of dysentery, while they are only judicious in a few exceptional cases of diarrhœa—astringents are seldom, or never, called for in dysentery, while they are nearly always appropriate in diarrhœa. It is no uncommon error for careless practitioners to confound dysentery with diarrhœa, and treat with powerful astringents, with the hopes of lessening the frequent desire to go to stool. We consider this a very grave error, and allude to the subject here, because teachers of medicine, more than all others, should draw a well-defined pathological and therapeutic line between the two diseases referred to.

*Raw Meat.*—In the *Charleston Medical Journal and Review* for March, Dr. F. P. Leverett has an article upon *raw meat* as a remedial agent. During the last year considerable has been said of it as a remedy in the diarrhœa of children. Most that we have seen upon the subject has been abstracted from foreign journals. Dr. Leverett says that Dr. Casper Morris introduced the use of raw beef into the children's ward of the Philadelphia Hospital, in the fall of 1855, and that he professed to have learned its powers over chronic diarrhœa of children from Prof. Thomas, of Baltimore; Dr. Leverett details a few interesting cases, occurring in the hospital under the care of Dr. Morris. The raw beef was not confined to the cases of children, but was equally beneficial in obstinate cases of chronic diarrhœa of adults. Dr. Leverett says he has found the remedy of much benefit in some other diseases. "In one case of chronic dyspepsia, with great irritability of the stomach, it was retained when almost everything else was rejected. In the latter stages of typhoid fever, it proved a valuable article of diet, as I should have mentioned that it had done at the Philadelphia Hospital."

*Quinine in Uterine Hæmorrhages.*—In the March number of the *Charleston Medical Journal and Review*, Dr. J. S. Rich, of Georgia, reports several cases of protracted uterine hæmorrhage of an alarming character, speedily relieved by the use of quinine, after the failure of all other known means. The following are his favorite methods of administration:

" ℞.—Sulph. quinine, ʒj.

Sulph. ferri, ʒj.

Mucil. gum Arabic, q. s. ut. ft.

Pillulæ, No. xxx.

℞.—Sulph. quinine, ʒj.

Sulph. ferri, ʒj.

Gum terebinth, ʒj.

M. ft. in mass. et. div. in pillulæ, No. xxx. S.—Two to be taken morning, noon, and night."

In the *Lancet and Observer* for March, one of the editors appends the following to an item taken from a former number of our *Summary:* "We recently attended a case of uterine hæmorrhage, in connection with Prof. Richardson, of this city, which assumed a regular intermittent type, and was cured by the free administration of quinine."

*Elephantiasis.*—In the *Charleston Medical Journal and Review*, Dr. T. L. Ogier, of Charleston, reports a case in which he ligated the femoral artery for the cure of elephantiasis of the leg and foot. The report was made about three months after the operation, at which time the result was quite satisfactory, the swelling having subsided. Dr. Ogier wisely says, that "at least a year must elapse before the disease can be said to be permanently eradicated." Prof. Carnochan has the honor of first proposing this operation. He has successfully operated four times. Prof. Erichsen, acting upon the suggestion of Prof. Carnochan, has once tied the anterior tibial artery in the middle of the leg, with results quite satisfactory.

*Hepatic Abscess.*—In the *New Orleans Medical News and Hospital Gazette* for March, Prof. Austin Flint has an article upon *suppurative inflammation of the liver.* Speaking of the escape of pus through the lungs, he says, "It is remarkable how little pulmonary inflammation is often excited under these circumstances. One would suppose, *à priori*, that pneumonia would be sure to be developed in the entire lung; and the fact that it does not, goes to show that the latter disease involves in its production, general, rather than local causes. Evacuation of an hepatic abscess through the lung appears to be a conservative mode. Recovery has been observed oftener when the pus is discharged in this direction than when the abscess points to the surface, and the perforation takes place through the integument. This, *à priori*, would certainly not be expected."

This opinion is contrary to the expressed opinion of most authorities; Drs. Budd, Copland, Watson, and such others with whose views upon this point we happen to be acquainted, think that the superficial discharge of pus is the most favorable way that the pus can make its escape. It is, however, proper to note here that Dr. John Jackson, in the October number of the American edition of the *London Lancet*, advocates opinions in accordance with those above quoted from Dr. Flint.

*Hydrocephalus!*—In the March number of the *New Orleans Medical News and Hospital Gazette*, Dr. Marsh, of Port Hudson, reports a very interesting case of hydrocephalus, in which *paracentesis capitis* was several times performed. At the time of the first operation the patient was about *nine* months old. "Its body and extremities were emaciated beyond the power of pen to describe. It seemed as though there were nothing but integuments, blood-vessels, and bones left. At the same time, it had the *face* of an infant and the head of a man." The head measured 26 inches in its longest diameter. At the first operation "eleven ounces of fluid were drawn off, clear and pellucid at first, and of a slightly saline taste, like perspiration, but turbid at last." Sixteen days later the second operation was performed, and *sixty-four* ounces were evacuated, "with a very strong smell of urine." Eleven days later, sixteen ounces of fluid were withdrawn, and three days later the patient died. Dr. Marsh says, "It may be proper here to observe, that at no time after an operation could the bones of the head be compressed so as not to leave a cavity between them and the brain. As the water again accumulated, it could be distinctly heard surging from one side to another, as the child was moved."

*Hæmaturia.*—In the *Medical and Surgical Reporter* for March 3d, Dr. E. T. Blackwell reports a case of hæmaturia, which full doses of gallic acid, opium, &c., failed to relieve. He says, "Finding the remedies entirely fail, I ordered the bladder to be injected with a weak solution of alum, at first tepid, afterwards entirely cold. The effect was happy and rapid."

*Scarlatina.*—In the *Medical and Surgical Reporter* for March 3d, Dr. Gebhard has a paper upon the treatment of scarlet fever, in which he speaks very highly of *digitalis* as a remedy. He reports quite a number of illustrative cases,ι n whicn the reported results are certainly quite satisfactory. For the last five years, during which he has used this remedy, he says he has lost but one case, and in that the remedy was brought to bear quite too late; " since that," he says, " as soon as any symptom indicated that the disease was scarlatina, or even a supposition to that effect, I have commenced the use of digitalis with a favorable result in every instance, as the cases related have fully confirmed." His method of administration was usually the following: for a patient from 4 to 6 years old, I ordered 40 grains of digitalis in 40 tea-spoonsful of hot water, and when cool, to take one tea-spoonful every hour, to be continued until the entire abatement of all the symptoms."

*Gelseminum.*—In the *Journal of Materia Medica* for March, Dr. A. F. Patte has an article upon the properties and uses of gelseminum. We quote only the following: "*Headache* of the nervous kind may often be relieved, and I have found no one medicine so useful in this troublesome disease. *Neuralgia*, in its various forms, may be treated with this remedy, both internally and externally, with the hope of benefit. In *coryza*, or cold in the head, this is one of the best remedies I have ever used; it cures the severest cases in from twelve to forty-eight hours."

*Indian Corn an Anti-Periodic.*—In the *Nashville Journal of Medicine and Surgery* Dr. J. W. Gambling, of Kentucky, has an article upon the use of corn-meal as a substitute for quinine. He says he has administered the corn-meal in intermittent fever " to thirty patients, with satisfactory results. It prevented the paroxysms in all but two cases, and I attributed the failure to the inferior quality of the article used." He says further, " I can state with certainty that corn-meal is an anti-periodic, and as near a specific in intermittents as quinia." We do not remember of having seen the corn-meal recommended as a medicinal agent of power, previous to seeing an extract from a letter from Dr. D. B. Phillips, of the United States Navy, which was published in the *N. A. Medico-Chirurgical Review* for Sept., 1858. He there recommended it highly in facial neuralgia. In the *American Journal of Medical Sciences* for October, 1858, he reports a case of intermittent fever, cured with the same agent. Our readers will remember that, in a former number of our *Summary*, we gave Dr. Nagle's views in regard to the pathology and treatment of *milk sickness*. For this disease he says, " that corn-meal is an anti-periodic and specific." Bearing upon the subject under consideration, we quote the following passage from Prof. G. S. Blackie's Introductory Lecture, published in the *Nashville Journal of Medicine and Surgery* for January of this year. "The specific for European chills has yet to be found. That for American chills is here. Our American *maize* cures our native chills, and where those chills are most abundant there most abundantly it grows." We have to regret that Prof. Blackie did not say more of this agent, of which he speaks so confidently.

*Opium an Antidote to Belladonna.*—Within the last few years considerable has been said in regard to the reciprocal antidotal powers of opium and belladonna. In the *N. A. Medico-Chirurgical Review* for March, Dr. A. Lopez reports a case of poisoning with belladonna, in which opium was successfully resorted to as a remedy. He says, "I was induced to test the reciprocal influential relation between the two poisons, and prescribed forthwith tinct. opii. f. ʒj. aq. cinnamon. ℥j. at one dose, with directions to repeat gtts. xv. every half hour, until a decided abatement of the pathological symptoms. The first dose antagonized the belladonna in less than thirty minutes after it was taken."

*Anæsthesia during Sleep.*—In the *Peninsular and Independent* for February, Dr. J. H. Beech has an article upon the above subject. At a meeting of the Buffalo Medical Association, several months ago, the possibility of chloroforming a person to insensibility during sleep was under discussion. It was the opinion of most of the members present that it was impossible thus to anæsthetize, without awaking the sleeper. Dr. Beech opposes this view, reports one case in illustration, and says he has succeeded in several other cases. He held a sponge as close to the nose and mouth as possible without touching, during inspiration, turned it aside when each expiration began." Complete anæsthesia was speedily induced. Dr. Beech says, "Persons sleeping will be sooner awakened by any article which resists the current of expiration, or turns it upon the face, than by offensive odors inhaled." The subject is one of interest, because of its medico-legal bearings.

*Wounds of the Scalp.*—In the *Southern Medical and Surgical Journal* for March, Prof. H. S. Campbell has a few remarks upon the dressing of wounds of the scalp. He proposes to avoid shaving the head, and also sutures. The plan which he advises, and has adopted, is that of "tying the strands of hair across the line of the wound, using them in the manner of sutures to effect approximation and retention of the edges." If the knot is disposed to slip, he says, "little *clamps of split shot* may be applied upon the strands of hair from the opposite sides." It appears to us that this plan is not new; yet Prof. Campbell says, he has "seen no record of it in recent works on surgery."

*Tonic for Dyspepsia.*—The *Lousville Medical News* for February gives the following for dyspeptic cases:

" ℞.—Tinct., cinchon.

" quassiæ, āā. f. ℥ij.

" nux vomicæ, f. ʒj. M.

A tea-spoonful three times a day in a wine-glassful of sweetened water. This is one of the best combinations of its kind; it is much prescribed by Dr. E. Wilson."

*Belladonna as an Antigallactic.*—In the *Medical and Surgical Reporter* for March 10th, Dr. John Flynn has an article upon the above subject. He says he has put the remedy to a rigid test, and entirely without benefit. In six cases, the most favorable for insuring a fair trial, he has " found it utterly worse than useless." This seems a little strange to us, for there is no remedy in any disease that has given us such gratifying results. We have previously given our experience in former numbers of our *Summary.* As our experience with this article is constantly accumulating, we still add such as has occurred to us since writing our last *Summary.* About two weeks since, we attended a primipara having a contracted pelvis. We attempted to deliver with forceps, but failing in this, we were compelled to resort to craniotomy. The following day we commenced the use of fluid extract of belladonna, locally applied to the breasts. The patient made a good recovery; no breast-pump was used, no nursing performed, and yet the breasts did not inflame. About ten days since we attended another primipara, in which the labor was easy and soon over, yet the child was still-born. On the following day we directed the fluid extract of belladonna to be applied to the breasts twice, daily, and oftener should symptoms of inflammation supervene. For a few days there was a small secretion of milk, which was drawn, but now the breasts are flaccid, having shown no evidences of inflammation. Every physician knows that it is in just such cases as these that mammary abscesses occur.

Since we commenced using the belladonna, perhaps three years since, we have not seen a case of mammary abscess, except in cases where mammary inflammation had been permitted to progress to near or quite the stage of suppuration, before we were called upon to advise in the cases. We certainly regard the belladonna as one of the greatest boons intrusted to us for the relief of that unfortunate class of females, who, after undergoing the pains of maternity, are compelled from any cause to forego the pleasures of nursing. It is proper to observe, that in the cases just referred to, we administered at the time the iodide of potassium, internally. How much effect the latter remedy may have had in securing the desired result, we do not know; certain are we that the combination of influences is apparently all that heart could wish in such cases. The preparation of belladonna used was Tilden & Co.'s fluid extract.

*Extra Uterine Pregnancy.*—In the *Chicago Medical Journal* for March, Dr. C. Goodbrake reports a very interesting case of extra uterine pregnancy. The patient had previously borne nine children. In the spring of 1856, she supposed herself pregnant for the tenth time. At the fifth month, fœtal movements were quite distinct. In December of the same year, pains occurring, her physician was summoned, and though pains continued for several hours, no child was born. Three weeks later, the physician was again called, with result as before. She continued to carry the child until the fall of 1859. Many physicians were consulted, and many opinions given, but no relief or quietude of mind obtained. On the 24th of October last, Dr. Goodbrake performed gastrotomy. The child was found enveloped in a sac of its own. "The sac was found firmly adherent in the right iliac fossa, and to a considerable extent, to the parietal peritoneum on the right side. There were no adhesions anteriorly, nor to the intestines, which were all crowded to the left side." The child was of the female sex, of medium size, as at full period, and presented no evidences of decomposition. It was removed, but the patient died on the fifth day after the operation.

*Variola and Vaccina.*—In the *Boston Med. and Surg. Journal,* for March 15th, Dr. Ephraim Cutter has an interesting article upon the above subjects. It has been the prevailing opinion of the profession, that the vaccine disease was small-pox, modified and mitigated by its transmission through the cow. The experiments of Dr. Cutter seem to negative this opinion. He and his associate in experiment, Dr. Alonzo Chapin, have repeatedly *inoculated* the cow with variolous poison, by various methods, and entirely without effect. They have *vaccinated* the cow with the effect of developing the characteristic pustule. Dr. Cutter concludes, that cow-pox *is not* modified small-pox, but that they are distinct diseases. If the Dr's opinions should prove to be correct, we hope we shall hear no more about the gradual decade of the protective power of the vaccine disease, because of its successive transmissions through the human system.

Dr. Cutter does not claim originality in these views, for he says, "Dr. Von Bibra distinctly says, that the cow-pox and the small-pox are two different diseases."

*Podophyllin and Leptandrin.*—In the *Cleveland Medical Gazette* for March, Prof. J. P. Kirtland has an article upon the properties and uses of the above-mentioned agents. He believes that mercurials are quite too frequently and indiscriminately used, and that a combination of podophyllin and leptandrin may often be substituted for that agent with benefit.

Though the Professor's article will give aid and comfort to that class of quacks who glory in the cognomen of *eclectic*, yet his experience is none the less worthy of regard. His reputation for accuracy of observation will entitle his statement to confidence and respect. The indications for the use of the compound under consideration are the same as for the mercurials. Dr. Kirtland says, "My usual prescription for a laxative and aperient, as an equivalent for one or two grains of calomel, or five grains of blue mass, is the following:

℞.—Podophyllin,
Leptandrin, ää, x grs.

Mix thoroughly, and divide into XL powders. Dose, one powder at bedtime; repeat, as occasion may require. Ale, coffee, or Catawba wine forms a convenient and palatable vehicle.

*Uterine Hæmorrhage.*—In the *Cleveland Medical Gazette* for March, Dr. Barth. Weber, of Cincinnati, has an article upon a new and sure method of arresting uterine hæmorrhage. The method consists in *compressing the arteria aorta descendens.* "In order to perform this small operation, the patient is to be placed on the back, the pelvis somewhat raised, and the thighs drawn up towards the abdomen, so that the abdominal wall gets relaxed; then you search with stretched hand for the fundus uteri, which, generally, you will find near the navel; push forward with the fore and middle finger, immediately above the fundus uteri, in a perpendicular direction, and at the same time try to push the intestines upward, which, generally, is easily accomplished. In this manner, you reach the spinal column, and feel plainly the pulsation of the abdominal artery. Now, you press with fore and middle finger perpendicularly, upon the artery, the hand forming almost a right angle with the spinal column." In this way, Dr. Weber says the artery can be compressed at pleasure. "This compression is to be continued as long as there is any danger to life from hæmorrhage, often for hours."

*Prolapsus of the Funis.*—In the *Louisville Medical Journal*, for March, Dr S. Branders has an article on the treatment of prolapsus of the funis. He reports *three* cases successfully treated by position. He places the patient upon her breast and knees, introduces his hand into the vagina or uterus, returns the cord, and retains his hand in position until the head is ready to occupy the entire pelvic strait. He ascribes success mainly to the position. Dr. Branders ascribes the original suggestion to Dr. Thomas, of New York. We are aware that Dr. T. G. Thomas read a paper upon this subject before the New York Academy of Medicine, very early in the year 1858. We also know that Prof. Mendenhall, of Cincinnati, has practiced replacement of the funis by position, for some time past; and that he has published two articles in the *Lancet and Observer* upon this subject, with illustrative cases. After the cord is replaced, so long as the patient will keep the position, it is entirely unnecessary to retain the hand in the vagina. The position should be kept until the head takes the position which will prevent the descent of the cord, when the woman should be placed upon the side or back during the balance of the labor.

*Injuries of the Skull.*—In the *Louisville Medical Journal* for March, Prof. Middleton Goldsmith has an article upon the *treatment of injuries of the skull*, in which he proposes a substitute for the *trephine*. It is an objectionable feature of the trephine that so much sound bone is necessarily sacrificed. To obviate this objection, Prof. Goldsmith has, for the last ten years, used the "*chisel in place of the former instrument.*" He says, "With a *chisel*, impelled by the hand, or small hammer, just so much bone may be removed as is necessary for the insertion of the elevator, or for the extraction of the detached pieces, and no more. The dura mater is not endangered in the operation, for the depressed bone protects it. It is never necessary to cut any part of the internal table, for if the opening in the external table is as large as the fracture of the internal table, then the external opening is large enough to allow the required extraction." To our mind, the chisel or gouge seems to possess such manifest advantages, that the only wonder is, that it had not long since suggested itself, and ere this, entirely superseded the trephine. Economy in the loss of skull bone is not a matter of trifling consideration.

Dr. Goldsmith says further, "In the next place, the writer has uniformly practiced the *immediate and perfect closure* of the wound in the scalp." He believes "that the safety of the patient is vested more in the exclusion of the atmosphere from contact with the dura mater than in any other thing, and in any other circumstance in the whole operation of trephining." . . . "Where there is no scalp wound, or when the latter is small, then the surgeon should make a semicircular flap, large enough to embrace the breach of bone, and to extend from onehalf to three-quarters of an inch beyond it." . . . "The incision should have no angles over the breach of the bone."

Dr. Goldsmith says he has operated for fracture of the skull, as above, more than twenty times during the last ten years, without losing a single case, and his colleague, Prof. Hardin, has operated seven or eight times also, without losing a case. This is certainly unprecedented success, and is, doubtless, in some measure related, as an effect, to the manner of operating and dressing the wound.

*Swallowing Teeth.*—In the *Dental Cosmos* for March, (an excellent dental journal, by the way,) Dr. Foster reports a remarkable accident of swallowing teeth. He says, "A gentleman of this city, (Wilmington,) thirty-five years of age, sanguine temperament, swallowed his artificial teeth at midnight on Wednesday. Physicians were called in, who fished for them, also tried the usual remedies, but all to no purpose; it was then concluded to let Nature take her course, (not doubting in the least that *her* course would be *death* to him,) when, to the astonishment of all, and his most unbounded delight, after a very painful and laborious stool, he found himself again in the possession of them. This did not take place until the following Monday, making the *round trip* in five days." The plate was quite heavy, to which three teeth were attached.

*Pneumonia, &c.*—In the first three numbers of the third volume of the *Medical Journal of North Carolina*, Dr. W. T. Howard has a very lengthy and able review of Dr O. F. Manson's essay on *malarial pneumonia.* We do not propose to enter the controversy, nor to give a synopsis of Dr. Howard's paper, which would well pay the perusal. We shall give only a brief notice of the treatment proposed. In malarial pneumonia, Dr. Howard favors the early and liberal use of quinine, and he enters with some minuteness into the literature of the treatment. He has, however, dealt more with early than with recent authors. Our readers are familiar with the fact, that we have, for some time, recommended quinine in pneumonia of an adynamic type, even though non-malarial. Our first article upon this subject was published in the *Lancet and Observer*, for October, 1858. In a former No. of our *Summary*, we have alluded to Dr. S. A. Cartwright's claim of first recommending quinine in large doses in pneumonia. He dates his claim as far back as 1826. Dr. Howard denies the claim of Dr. Cartwright, and avers that the quinine treatment was practiced, in some instances, both at home and abroad, anterior to that time. It matters but little, practically, who originated the treatment proposed; it should be universally known that, in *malarial pneumonia,*

quinine should constitute the principal treatment. Because of our ignorance and limited reading, we once supposed we were the first to advise quinine in the first stage of some forms of pneumonia, even when unconnected with a malarious influence. We have since learned that what was new to us was not so to some others. We believe that quinine is too generally regarded as exclusively a tonic. We have often expressed the opinion, that under some circumstances, it was a powerful *sedative.* It is to urge this opinion that we write the present article. Dr. Howard copies the opinions of Dr. G. A. Wilson, of North Carolina, upon this point, and they correspond so exactly with our own, that we quote them here: "*As early as the year* 1838, I had to unlearn all that had been taught me of this agent as a stimulant and tonic, and of the dangers attending its administration in inflammatory states of the system. I have often noticed its effects in that class of cases complicated by cerebral determinations, and can safely say I have never known injurious consequences to follow. If stimulant at all, it has not acted in my hands as stimulants are wont to do. In many cases of high nervous excitability, it has had soothing and *sedative* effects."

*Valerinate of Strychnia.*—In the *Medical Journal of North Carolina,* for March, Dr. R. Wysong has an article upon the above compound.

The following is his formula of preparation:

" ℞.—Sulph. strych., gr. viij.
Valerian. acid., ℥j. M."

He says: " I have been using the val. strych. some ten months, and find that it is more particularly adapted to those cases where there is general debility, accompanied with nervous excitability, loss of appetite, indigestion, constipation, depression of spirits, and all the symptoms following, more or less, on the want of tone in the nervous system."

*Treatment of Gonorrhœa.*—In the *Med and Surg. Reporter* for March 24th, Dr. A. H. Stephens has an article on the treatment of gonorrhœa with the extract of *conium maculatum.* He reports prompt and happy effects from this agent—the cure being complete in from three to eight days. He gives the extract in twelve-grain doses every two hours. If giddiness is produced, the dose is diminished.

*Ovariotomy.*—In the *Boston Medical and Surgical Journal* for March 29th, Dr. A. B. Crosby reports a successful case of ovariotomy. The patient was 36 years of age, married, the mother of two children. The tumor was first observed about five and a half years previously. Paracentisis had been several times performed, and fluid to the amount of 475 pounds had been drawn off. The operation was performed on the 28th of last August. The tumor and its contents weighed 28 pounds. Five weeks after the operation, the patient was able to dress herself, and direct her household affairs. There is no operation in which we take more interest than in ovariotomy. Several successful cases have been reported during the last year. Have the unsuccessful cases been suppressed?

*Diphtheria.*—In the *Lancet and Observer* for March, Dr. Isaac Meranda has a few remarks upon diphtheria. Dr. Meranda says: "A favorite remedy with us, and one which we consider admissible in every stage of the disease, is chlorate of potassa, combined with hydrochloric acid:

| | |
|---|---|
| " ℞.—Chlorat. potas. pulvis, | ʒij. |
| Hydrochloric. acid., | f. ʒj. |
| Aquæ, | f. ℥viij. M." |

. . . " Of this, half an ounce may be given every two or three hours, according to the urgency of the symptoms." This is not an original prescription; it was, so far as we know, first proposed and highly extolled by Mr. Lambden, of Coningsby, in the *London Lancet*, for November 20th, 1858.

In a plethoric patient, Dr. Meranda prescribes, at first, calomel and jalap, in full purgative doses. In more feeble patients, he gives " calomel in alterative doses, conjoined with opium and ipecacuanha, or with camphorated Dover's powders." . . . " In some chronic cases, I have seen the happiest effects follow a moderate salivation."

We do not believe in the utility or propriety of this liberal administration of calomel in a disease so characterized by early prostration. Of all the local applications for the throat, internally, he says: " We have found nothing equal to the nitrate of silver. We prefer the solid stick, when we can reach the affected part; when we cannot do this, we apply a strong solution by means of the probang."

In the last four weeks we have seen a few cases of diphtheria, of no mild type. We may report our experience hereafter.

